# Occurrence of NDM-1, VIM-1, and OXA-10 Co-Producing *Providencia rettgeri* Clinical Isolate in China

**DOI:** 10.3389/fcimb.2021.789646

**Published:** 2022-01-03

**Authors:** Siquan Shen, Xiangning Huang, Qingyu Shi, Yan Guo, Yang Yang, Dandan Yin, Xun Zhou, Li Ding, Renru Han, Hua Yu, Fupin Hu

**Affiliations:** ^1^ Institute of Antibiotics, Huashan Hospital, Fudan University, Shanghai, China; ^2^ Key Laboratory of Clinical Pharmacology of Antibiotics, Ministry of Health, Shanghai, China; ^3^ Department of Laboratory Medicine, Sichuan Provincial People’s Hospital, University of Electronic Science and Technology of China, Chengdu, China

**Keywords:** *Providencia rettgeri*, *bla*_NDM-1_, *bla*_VIM-1_, *bla*_OXA-10_, Mobile gene elements

## Abstract

*Providencia rettgeri* is a nosocomial pathogen associated with urinary tract infections related to hospital-acquired Infections. In recent years, *P. rettgeri* clinical strains producing New Delhi Metallo-β-lactamase (NDM) and other β-lactamase which reduce the efficiency of antimicrobial therapy have been reported. However, there are few reports of *P. rettgeri* co-producing two metallo-β-lactamases in one isolate. Here, we first reported a *P. rettgeri* strain (P138) co-harboring *bla*_NDM-1_, *bla*_VIM-1_, and *bla*_OXA-10_. The specie were identified using MALDI-TOF MS. The results of antimicrobial susceptibility testing by broth microdilution method indicated that *P. rettgeri* P138 was resistant to meropenem (MIC = 64μg/ml), imipenem (MIC = 64μg/ml), and aztreonam (MIC = 32μg/ml). Conjugation experiments revealed that the *bla*_NDM-1_-carrying plasmid was transferrable. The carbapenemase genes were detected using PCR and confirmed by PCR-based sequencing. The complete genomic sequence of the *P. rettgeri* was identified using Illumina (Illumina, San Diego, CA, USA) short-read sequencing (150bp paired-end reads), and many common resistance genes had been identified, including *bla*_NDM-1_, *bla*_VIM-1_, *bla*_OXA-10_, *aac(6’)-Il, aadA5, ant(2’’)-Ia, aadA1, aac(6’)-Ib3, aadA1, aph(3’)-Ia, aac(6’)-Ib-cr*, *qnrD1*, *qnrA1*, and *catA2*. The *bla*_NDM-1_ gene was characterized by the following structure: IS*110*–TnpA–IntI1–aadB–IS*91*–GroEL–GroES–DsbD–PAI–ble–*bla*_NDM-1_–IS*91*–QnrS1–IS*110*. Blast comparison revealed that the *bla*_NDM-1_ gene structure shared >99% similarity with plasmid p5_SCLZS62 (99% nucleotide identity and query coverage). In summary, we isolated a *P. rettgeri* strain coproducing *bla*_NDM-1_, *bla*_VIM-1_, and blaOXA-10. To the best of our acknowledge, this was first reported in the world. The occurrence of the strain needs to be closely monitored.

## Introduction

*Providencia rettgeri* is an opportunistic human pathogen, unlike other *Enterobacterales*, it is a little-known pathogen, which is mainly associated with hospital-acquired infections including catheter-related urinary tract infections, bacteremia, meningitis, diarrhea, and eye infections (Yoh et al., 2005; Tada et al., 2014). Treatment of these infections is challenging, as they are intrinsically resistant to multiple antibiotics including first-generation cephalosporins, amoxicillin-clavulanic acid, nitrofurantoin, tigecycline, and polymyxins. Imipenem, amikacin, and cefepime are effective against more than 90% of the isolates (Lee et al., 2007; Sharma et al., 2017). However, in recent years *P. rettgeri* has become increasingly carbapenemase producers carrying the carbapenem-resistant genes like *bla*_NDM_, *bla*_VIM_, and so on (Piza-Buitrago et al., 2020). The emergence of multidrug-resistant of *P. rettgeri* strains poses a serious threat to public health.

The widespread of metallo-β-lactamases (MBLs) remain a severe challenge in health care settings because the hydrolysis of β-lactams by MBL enzymes cannot be prevented by clinically available β-lactamase inhibitors, including avibactam, relebactam, and vaborbactam (Wu et al., 2019). New Delhi Metallo-β-lactamases were the most predominant MBL among *Enterobacterales* clinical isolates which were initially identified in *Klebsiella pneumoniae* in 2009 in a Swedish patient (Pillai et al., 2011). Currently, although *bla*_NDM-1_ was commonly related to *K. pneumoniae* (Han et al., 2020), *E. coli*, *Enterobacter cloacae*, and *Citrobacter freundii* strains in China (Yong et al., 2009; Zhang et al., 2021), reports on *bla*_NDM-1_ producing *P. rettgeri* are rare. The spread of plasmid-bearing MBL possess a great challenge for clinical treatment because these multidrug-resistant isolates will result in limitations on treatment options (Oteo et al., 2014; Piza-Buitrago et al., 2020). Here, we report the co-existence of the carbapenemase genes *bla*_NDM-1_, *bla*_VIM-1,_ and *bla*_OXA-10_ in a *P. rettgeri* clinical isolate in China.

## Materials and Methods

### Species Identification, Antimicrobial Susceptibility Testing, and Confirmation of Carbapenemase Production

Species identification was performed using MALDI-TOF MS (bioMérieux, France). The minimal inhibitory concentration (MIC) was determined by the broth microdilution method according to the guidelines of the Clinical Laboratory Standards Institute (CLSI) (Clinical and Laboratory Standards Institute, 2021). The strains *E. coli* ATCC 25922 and *Pseudomonas aeruginosa* ATCC 27853 were used as quality controls for antimicrobial susceptibility testing. Quality control and interpretation of the results were based on 2021 CLSI breakpoints (CLSI, 2021) for all the antimicrobial agents with the exception of cefepime-tazobactam, tigecycline, and polymyxin B. Cefepime- tazobactam MICs were interpreted using CLSI breakpoints for cefepime for comparison purposes only. Tigecycline and polymyxin B MICs were interpreted using the European Committee for Antimicrobial Susceptibility Testing (EUCAST) criteria (EUCAST, 2021). Carbapenemase production was phenotypically detected using imipenem- 3-aminobenzeneboronic acid/EDTA double disk synergy test. The existence of the carbapenemase genes (KPC, NDM, OXA, IMP, and VIM) was confirmed by NG-Test Carba-5 and PCR-based sequencing, as previously described (Poirel et al., 2011b; Weiß et al., 2017).

### Conjugation Assay and Plasmid Sequencing

Conjugation experiments were performed to explore the transferability of the plasmid using azide-resistant *E. coli* J53 as a recipient strain. The conjugants were selected on Mueller-Hinton (MH) agar supplemented with azide (100 mg/L) and ampicillin (50 mg/L). The conjugation frequency was calculated according to the number of conjugants per initial donor bacteria. The presence of the *bla*_NDM-1_, *bla*_VIM-1_, *bla*_OXA-10_ in conjugants was confirmed by PCR and PCR-based sequencing. The Qiagen Midi kit (Qiagen, Hilden, Germany) was used to extract the plasmid of the conjugant and the plasmid was sequenced using Illumina (Illumina, San Diego, CA, USA) short-read sequencing (150bp paired-end reads). SPAdes 3.12.0 was used to *de novo* assemble the sequencing reads, and the open reading frame prediction and annotation were done with RAST version 2.0 (https://rast.nmpdr.org) and BLAST (https://blast.ncbi.nlm.nih.gov/Blast.cgi). The plasmid replicon was determined using the PCR-based replicon typing method (Carattoli et al., 2005). Plasmid comparisons were performed using BRIG (http://brig.sourceforge.net) (Alikhan et al., 2011) and Easyfig tools (http://mjsull.github.io/Easyfig) (Sullivan et al., 2011). Plasmids carrying *bla*_NDM-1_ were circularized using PCR and Sanger sequencing to fill in gaps between contigs. The conjugation elements were detected using oriTfinder, a web-based tool for the identification of origin of transfers in DNA sequences of bacterial mobile genetic elements (https://tool-mml.sjtu.edu.cn/oriTfinder/oriTfinder.html) (Li et al., 2018).

### Whole Genome Sequencing and Bioinformatics Analysis

The isolates’ genomic DNA was obtained by using one commercial kit, according to the manufacturer’s recommendation: Qiagen for P138. And the genomic DNA was sequenced using Illumina (Illumina, San Diego, CA, USA) short-read sequencing (150bp paired-end reads). Reads were trimmed with sickle (GitHub), subsequently, they were *de novo* assembled using SPAdes 3.12.0. Antimicrobial resistance genes analysis was performed using BacWGSTdb (http://bacdb.cn/BacWGSTdb/analysis_single.php) and the annotation process was done using RAST version 2.0 (https://rast.nmpdr.org).

## Results

### Overview of the *P. rettgeri* Clinical Isolate

The *P. rettgeri* strain P138 was isolated from a 51-year-old female patient that was admitted to a public hospital for the treatment of cervical cancer in 2019 in Sichuan Province in the southwest of China. A hysterectomy was performed for this patient. At the same time, due to the dense adhesion between the patient’s bilateral ureters and the paravaginal tissue, stents were put in the bilateral ureters. On the day before the operation, cefathiamidine (2g Q8h) was used for seven days for prophylaxis. On the ninth day after the operation, the patient developed a fever, an *E. coli* and the *P. rettgeri* strain P138 were isolated from urine culture, therapeutic regimen switched to levofloxacin (0.5g QD) and ceftizoxime (2g Q12h) for 2 days. Two days later, the patient’s body temperature returned to normal and the infection was controlled. Finally, the patient recovered and was discharged successfully.

The antimicrobial susceptibility profiles of *P. rettgeri* P138 are presented in **Table 1**. The isolate was resistant to all tested antimicrobial agents including amikacin (MIC >128μg/ml), cefoperazone-sulbactam (MIC >128μg/ml), aztreonam (MIC = 32μg/ml), piperacillin-tazobactam (MIC ≥256μg/ml), meropenem (MIC =64μg/ml), imipenem (MIC =64μg/ml), ceftazidime-avibactam (MIC ≥64μg/ml), tigecycline (MIC = 2μg/ml), and polymyxin B (MIC > 16μg/ml).

**Table 1:** Susceptibility of *P. rettgeri* clinical isolate, conjugant, and recipient to antimicrobial agents.

Strains	β-Lactamase genes	MIC (mg/liter)														
		CZA	IPM	MEM	CAZ	FEP	TZP	CSL	ATM	AMK	FPT	SXT	LEV	CIP	TGC	POL
*P. rettgeri* P138	bla_NDM-1_, bla_VIM-1_ and bla_OXA-10_	>32	64	64	>32	128	>256	>128	32	>128	>64	>32	>16	>8	2	>16
*E. coli* P138-C	bla_NDM-1_	>32	2	8	>32	32	>256	>128	≤1	2	32	0.25	0.5	0.5	0.125	0.25
*E. coli* J53	–	0.5	0.25	≤0.03	0.5	≤0.06	4	≤1	≤1	≤1	≤0.03	≤0.25	0.125	≤0.06	0.125	0.25

CZA, ceftazidime-avibactam; IPM, Imipenem; MEM, meropenem; CAZ, ceftazidime; FEP, cefepime; TZP, piperacillin-tazobactam; CSL, cefoperazone-sulbactam; ATM, aztreonam; AMK, amikacin; FPT, Cefepime-tazobactam; SXT, trimethoprim-sulfamethoxazole; LEV, levofloxacin; CIP, ciprofloxacin; TGC, tigecycline; POL, polymyxin B.

### Carbapenemase Genes and Conjugation Experiments

PCR-based sequencing demonstrated the presence of *bla*_NDM-1_, *bla*_VIM-1,_ and *bla*_OXA-10_ in *P. rettgeri* strain P138. According to the results of Conjugation Experiments, conjugants were positive for *bla*_NDM-1_ but negative for *bla*_VIM-1_ and *bla*_OXA-10_, making the conjugants resistant to meropenem (MIC = 8μg/ml) and ceftazidime-avibactam (MIC = >32μg/ml), intermediate to imipenem (MIC = 2μg/ml). The meropenem, imipenem, and ceftazidime-avibactam MICs of conjugants increased at least 256, 8, 128-fold respectively, compared with the recipient *E. coli* J53 (**Table 1**). The conjugation frequency is 2.47 × 10^–5^ (The conjugation frequency was calculated according to the number of conjugants per initial donor bacteria). Lots of modules associated with conjugation were detected in pP138-NDM, like the oriT gene (origin of transfer gene), relaxase, type IV coupling protein (TraD), and type IV secretion system (T4SS).

### WGS Analysis and Characterization of Plasmid Sequence Carrying *bla*_NDM-1_ Gene

According to the whole-genome sequencing analysis, many common resistance genes had been identified, including the carbapenemase genes *bla*_NDM-1_, *bla*_VIM-1_ and *bla*_OXA-10_, the aminoglycoside resistance genes *aac(6’)-Il, aadA5, ant(2’’)-Ia, aadA1, aac(6’)-Ib3, aadA1, aph(3’)-Ia* and *aac(6’)-Ib-cr*, the fluoroquinolone resistance genes *qnrD1* and *qnrA1* and the phenicol resistance gene *catA2*. The sequencing of the conjugant’s plasmid localized *bla*_NDM-1_ on a plasmid of 120,528 bp, belonging to the IncC type. Four resistance genes were identified in the plasmid pP138-NDM, *bla*_NDM-1_, *qnrA1*, *sul1*, and *ant(2’’)-Ia*, conferring resistance to carbapenems, quinolones, sulphonamides, and aminoglycosides, respectively. BLAST comparison disclosed that the *bla*_NDM−1_ gene environment of the plasmid pP138-NDM shared >99% similarity with plasmid p5_SCLZS62 (99% nucleotide identity and query coverage), isolated from a *Raoultella planticola* strain from Sichuan, China (GenBank accession number CP082173). In both plasmids, *bla*_NDM-1_ and *qnrA1* were located in an identical multidrug resistance region (MRR). The MRR was flanked by genes of IS*110* family transposase on both sides, and also contained IS*91*. Tn*As3*, which belongs to the Tn*3* family was also found in pP138-NDM. The full genetic environment surrounding *bla*_NDM-1_ is: IS*110*–TnpA–IntI1–aadB–IS*91*–GroEL–GroES–DsbD–PAI–ble–*bla*_NDM-1_–IS*91*–QnrS1–IS*110*.

**Figure 1:**
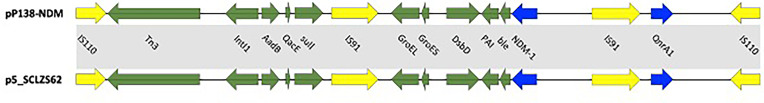
Circular comparison between plasmid pP138-NDM (MZ670000) and other similar plasmids. Plasmid pP138-NDM (the outer circle) was used by the BRIG software as a reference plasmid to perform the sequence alignment with BLASTN. The different colors indicate different plasmids and are listed in the color key.

In several plasmids with similar sequences (**Figure 1**), pSAL-19-0623_NDM (99% nucleotide identity and query coverage), an IncA/C2-type *bla*_NDM-1_ carrying plasmid with 276,695 bp in a carbapenem-resistant *Salmonella* strain from Singapore (GenBank accession numbers NZ_CP020913) (Octavia et al., 2020). They all showed resistance to meropenem and ceftazidime/avibactam, the only difference is that the strain P138 in our study was resistant to aztreonam, and this is most likely mediated by *bla*_OXA-10_.

## Discussion

The first isolate of NDM-1 producing *P. rettgeri* was reported in Israel in 2013 (Gefen-Halevi et al., 2013). Since then, NDM-1-producing *P. rettgeri* has been reported in various parts of the world (Pillai et al., 2011; Barrios et al., 2013; Carvalho-Assef et al., 2013; Mataseje et al., 2014; Pasteran et al., 2014; An et al., 2016). Reports in Nepal (Tada et al., 2014) as well as reports in Colombia (Marquez-Ortiz et al., 2017; Piza-Buitrago et al., 2020), and Korea (Shin et al., 2018), commonly associate *P. rettgeri* with high resistance rates to carbapenems. This resistance characteristic in *P. rettgeri* is commonly associated with the production of *bla*_NDM-1_. Recently, Piza-Buitrago et al. reported two NDM-1, VIM-2, and OXA-10 coproducing *P. rettgeri* strains GMR-RA257 and GMR-RA1153, similar to the drug resistance spectrum in our study, with resistance to the carbapenems imipenem and meropenem, and this was highly probable caused by the production of NDM-1 and VIM-1 (Piza-Buitrago et al., 2020). However, as to OXA-10, it seemed to have a limited effect on the hydrolysis of carbapenems, according to a study in Nigeria in 2011, a *P. rettgeri* isolate co-producing *bla*_OXA-10_, *bla*_VEB-1,_ and *bla*_CMY-4_ genes with no presence of the MBL genes was susceptible to carbapenems (Aibinu et al., 2011).

**Figure 2:**
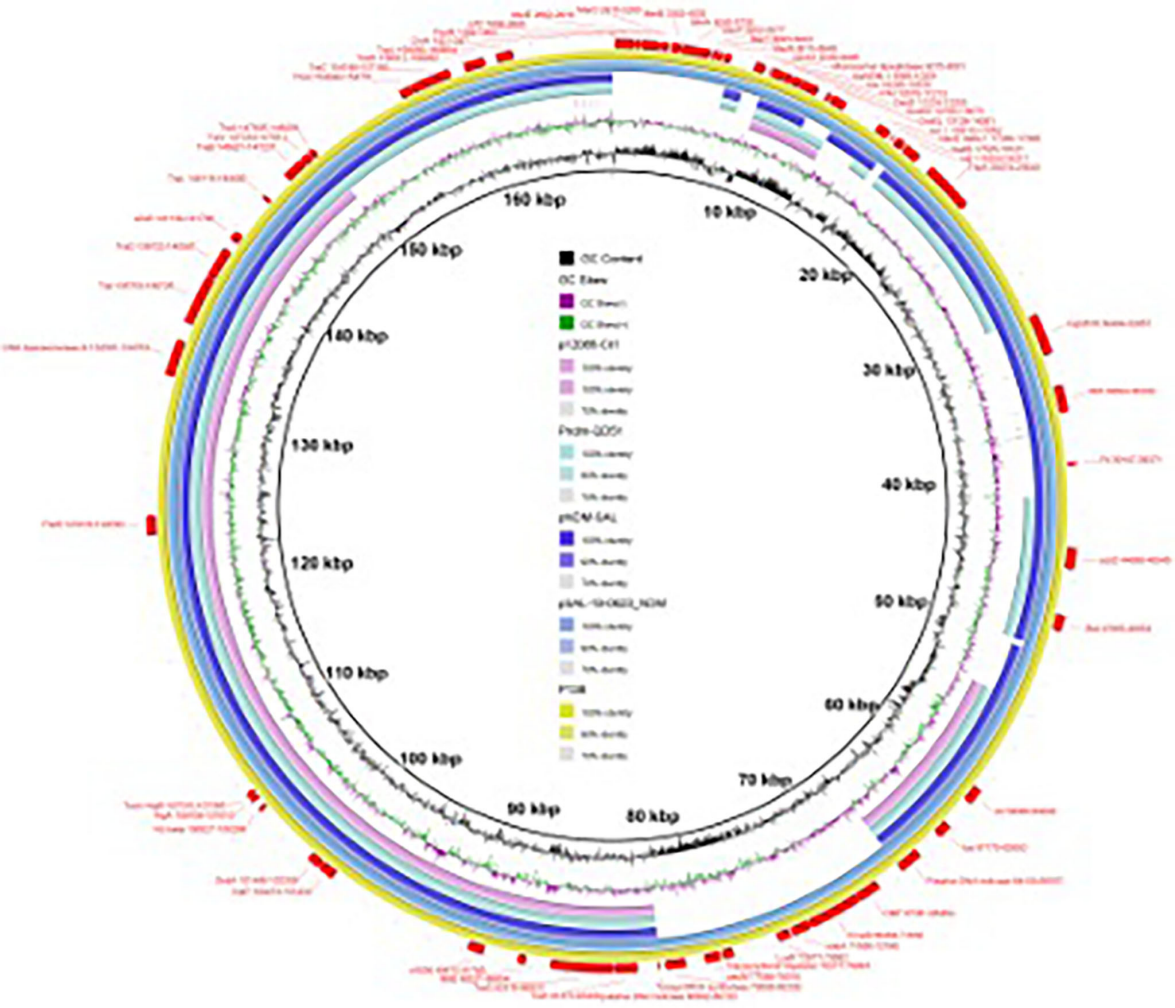
Multidrug resistance region (MRR) of *bla*_NDM-1_ in the plasmid pP138-NDM (MZ670000) and p5_SCLZS62 (CP082173). Resistance genes are indicated by blue symbols. Transposon-related genes and insertion sequences are indicated by yellow symbols. Other genes are indicated by violet symbols. Light gray shading indicated homologous regions (>99% DNA identity).

The moving elements can aggregate and combine with resistance genes, resulting in multiple resistance transfer of plasmids (Partridge, 2011). Different from previous studies often associated *bla*_NDM-1_ with Tn*125*, especially IS*Aba125* (Nordmann et al., 2011; Poirel et al., 2011a; Nordmann et al., 2012), in our study, was Tn*As3*, which is relatively rare reported. As to insertion sequences, IS*26* is widely distributed and it is often combined with Tn*125* family transposons (Poirel et al., 2011a; Zheng et al., 2021), in *P. rettgeri* isolate P138, *bla*_NDM-1_ was associated at its 3’-end and 5’ -end with IS*110* that is also relative rare reported. This further reflects the diversity of genetic elements, which leads to the wide spread of resistance genes among bacteria. Gene encoding small multidrug resistance (SMR) efflux transporter was also found in the MRR, such transmembrane proteins were frequently found in Gram-negative and Gram-positive bacteria where they were deduced to be associated with the efflux system (Kazama et al., 1998) (**Figure 2**).

In our study, we reported a carbapenem resistant *P. rettgeri* isolate P138, co-harboring *bla*_NDM-1_, *bla*_VIM-1_, and *bla*_OXA-10_, combined with the previous results (Pillai et al., 2011; Barrios et al., 2013; Carvalho-Assef et al., 2013; Mataseje et al., 2014; Pasteran et al., 2014; Tada et al., 2014; An et al., 2016; Marquez-Ortiz et al., 2017; Piza-Buitrago et al., 2020), the presence of MBL genes as *bla*_NDM-1_, *bla*_VIM-1,_ and *bla*_VIM-2_ contribute significantly to carbapenem resistance in *P. rettgeri*, while *bla*_OXA-10_ plays a relatively weak role. With the increasing number of such multi-drug resistant bacteria, especially these showed resistance to carbapenems like imipenem, meropenem, and new combination of antimicrobials like ceftazidime-avibactam, the clinical treatment options are limited, so the initial effective anti-infection treatment is important to reduce the mortality of infection caused by CRE. In the future, the laboratory should strengthen the monitoring of carbapenemase, and perform combined antimicrobial susceptibility tests to seek an effective therapeutic regime for the infection caused by CRE strain.

## Data Availability Statement

The datasets presented in this study can be found in online repositories. The names of the repository/repositories and accession number(s) can be found below: https://www.ncbi.nlm.nih.gov/, MZ670000.

## Ethics Statement 

The study protocol was approved by the Institutional Review Board of Huashan Hospital, Fudan University (Number: 2018-408).

## Author Contributions

FH and HY designed the study. SS and XH collected clinical samples and performed the experiments. SS, LD, YY, RH, QS, DY, YG, and XZ analyzed data. SS wrote the manuscript. All authors contributed to the article and approved the submitted version.

## Funding

This work was supported by the National Natural Science Foundation of China (81871690, and 81861138051), and Shanghai Public Health System Construction Three-Year Action Plan (2020-2022), Discipline leader Grant (GWV-10.2-XD02). The funders had no role in study design, data collection, and analysis, decision to publish, or preparation of the manuscript.

## Conflict of Interest

The authors declare that the research was conducted in the absence of any commercial or financial relationships that could be construed as a potential conflict of interest.

## Publisher’s Note

All claims expressed in this article are solely those of the authors and do not necessarily represent those of their affiliated organizations, or those of the publisher, the editors and the reviewers. Any product that may be evaluated in this article, or claim that may be made by its manufacturer, is not guaranteed or endorsed by the publisher.
